# Identification of Lactoferricin B Intracellular Targets Using an *Escherichia coli* Proteome Chip

**DOI:** 10.1371/journal.pone.0028197

**Published:** 2011-12-02

**Authors:** Yu-Hsuan Tu, Yu-Hsuan Ho, Ying-Chih Chuang, Po-Chung Chen, Chien-Sheng Chen

**Affiliations:** 1 Graduate Institute of Systems Biology and Bioinformatics, National Central University, Jhongli City, Taiwan; 2 Department of Food Science, National Taiwan Ocean University, Keelung City, Taiwan; Cairo University, Egypt

## Abstract

Lactoferricin B (LfcinB) is a well-known antimicrobial peptide. Several studies have indicated that it can inhibit bacteria by affecting intracellular activities, but the intracellular targets of this antimicrobial peptide have not been identified. Therefore, we used *E. coli* proteome chips to identify the intracellular target proteins of LfcinB in a high-throughput manner. We probed LfcinB with *E. coli* proteome chips and further conducted normalization and Gene Ontology (GO) analyses. The results of the GO analyses showed that the identified proteins were associated with metabolic processes. Moreover, we validated the interactions between LfcinB and chip assay-identified proteins with fluorescence polarization (FP) assays. Sixteen proteins were identified, and an *E. coli* interaction database (EcID) analysis revealed that the majority of the proteins that interact with these 16 proteins affected the tricarboxylic acid (TCA) cycle. Knockout assays were conducted to further validate the FP assay results. These results showed that phosphoenolpyruvate carboxylase was a target of LfcinB, indicating that one of its mechanisms of action may be associated with pyruvate metabolism. Thus, we used pyruvate assays to conduct an *in vivo* validation of the relationship between LfcinB and pyruvate level in *E. coli*. These results showed that *E. coli* exposed to LfcinB had abnormal pyruvate amounts, indicating that LfcinB caused an accumulation of pyruvate. In conclusion, this study successfully revealed the intracellular targets of LfcinB using an *E. coli* proteome chip approach.

## Introduction

The human immune system is divided into innate and adaptive systems. The innate immune system defends the host from infections in a non-specific fashion. This system exists in all animals and plants and is the first line of defense against invading organisms. One of the most important elements of the innate immune system is the antimicrobial peptide.

Natural antimicrobial peptides are pervasive in plants, insects and animals. They have been demonstrated to kill or inhibit the growth of bacteria, fungi, cancer cells and viruses [1]–[3]. LfcinB is a cationic antimicrobial peptide consisting of 25 amino acids that has been found to be present in many mammals for innate immunity. Similar to other cationic antimicrobial peptides [4], LfcinB exerts its antimicrobial activities via a pore-forming mechanism [5]. LfcinB also inhibits bacterial growth by impeding intracellular activities without destroying membrane integrity or penetrating the cytoplasmic membrane [6]. Ulvatne *et al.* [7] further observed that LfcinB at a sublethal concentration inhibited DNA, RNA and protein synthesis of *E. coli* and induced filamentation during an SOS-response in bacteria. These results suggest that LfcinB has multiple intracellular targets in bacteria.

Although several reports have observed that LfcinB inhibited intracellular activities in bacteria, the intracellular targets of LfcinB are still unknown. Thus, a high-throughput platform is needed to globally identify the interactions between LfcinB and the entire proteome. A proteome chip, also known as a proteome microarray, contains most of the individually purified proteins in a given proteome and is one potential platform that allows high-throughput identification of protein interactions in a single experiment [8], [9]. Proteome chips have been used to discover protein-DNA, protein-lipid, protein-drug and protein-peptide interactions [10], [11], and have become a powerful technology for systems biology [12]. In addition, proteome chip assays, which can be completed in hours, are faster and simpler than other proteomic tools [13]. Several proteome chips have been fabricated, including yeast, *E. coli* and human [8], [9], [14]. Chen *et al.* [9] developed a high-throughput protein expression and purification protocol to fabricate an *E. coli* proteome chip and applied it to the discovery of DNA damage-recognition activities. Moreover, *E. coli* proteome chips were used to identify serological biomarkers for inflammatory bowel disease [15] and screened for the substrates of the protein acetyltransferase [16]. These results demonstrated that the *E. coli* proteome chip is a powerful tool to globally identify the interacting proteins of certain molecules.

**Figure 1:**
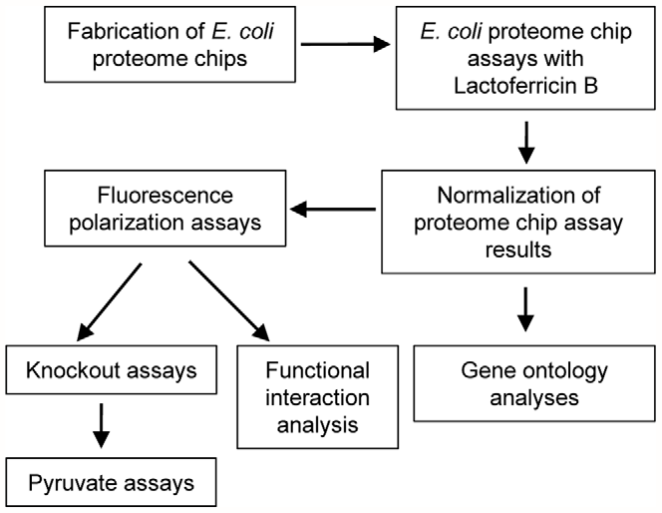
Scheme for the identification of intracellular targets of LfcinB. We used a high-throughput protein purification protocol to purify ∼4,300 *E. coli* proteins. These proteins were printed onto Fullmoon slides to fabricate *E. coli* proteome chips. We then probed LfcinB with *E. coli* proteome chips and analyzed the chip assay results by bioinformatics analyses. FP assays were then used to validate the identified proteins and 16 proteins were confirmed to be bound to LfcinB. We conducted knockout assays to further validate the 16 proteins and the EcID was used to observe the functional interactions of these 16 proteins. The knockout assay results revealed that one of the mechanisms of LfcinB action was associated with pyruvate metabolism. Finally, we used pyruvate assays to observe the relationship between LfcinB and pyruvate.

Herein, we utilized the *E. coli* proteome chip to identify the intracellular targets of LfcinB. The overall scheme for this identification method is shown in Figure 1. A novel feature of this methodology is the use of a high-throughput platform to begin this study and GO was used to analyze the targets of LfcinB. The identified proteins were then validated by FP assays. These results were not only analyzed by the EcID but also further validated by knockout assays. The results showed that one of the mechanisms of LfcinB action was highly related to pyruvate metabolism. Finally, we conducted pyruvate assays to confirm the influence of LfcinB on pyruvate levels in *E. coli*.

## Results

### *E. coli* proteome chip assays

**Figure 2:**
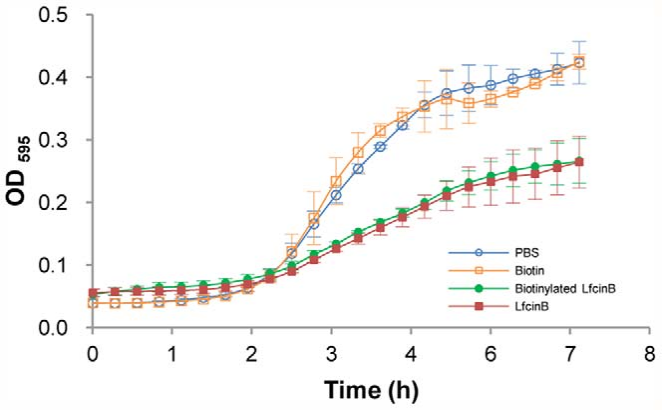
The antibacterial activities of biotinylated LfcinB, LfcinB and biotin. To confirm whether biotinylated LfcinB has different antibacterial activity than unbiotinylated LfcinB, we measured the growth curves of *E. coli* with each of them. The growth of *E. coli* with free biotin or with PBS, as a negative control, was also compared. The growth curves of *E. coli* with biotinylated LfcinB or LfcinB exhibit the same antibacterial activity. *E. coli* with biotin is similar to the negative control.

In this study, we incubated LfcinB with *E. coli* proteome chips to identify LfcinB-binding proteins. To provide the signal for LfcinB in *E. coli* proteome chip assays, LfcinB was labeled with biotin. To confirm that the biotinylated LfcinB used in the chip assays has the same antimicrobial activity as unbiotinylated LfcinB, biotinylated LfcinB, LfcinB and biotin were tested separately for their *E. coli* inhibition ability. As shown in Figure 2, LfcinB and biotinylated LfcinB showed similar growth curves and exhibited the same inhibition of *E. coli* growth; however, the growth curve of *E. coli* with biotin was similar to that of *E. coli* with phosphate-buffered saline (PBS). These results suggest that biotin-labeled LfcinB retains the antimicrobial activity of LfcinB. Moreover, free biotin had no influence on *E. coli* growth.

**Figure 3:**
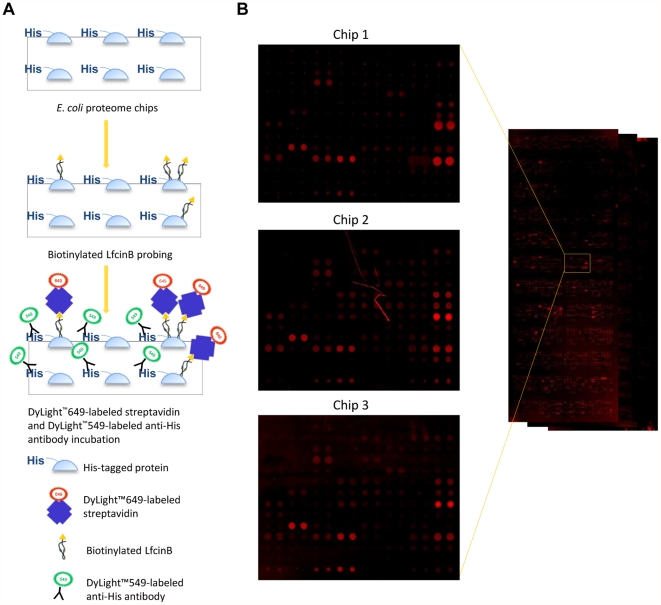
*E. coli* proteome chip assays with LfcinB. **A.** Schematic of chip assays. To select the proteins bound by LfcinB, *E. coli* proteome chips were incubated with biotinylated LfcinB. DyLight™ 649-labeled streptavidin was then probed with chips to identify the targets of LfcinB. DyLight™ 549-labeled anti-His antibody was used to detect the relative amount of the proteins. Each protein was printed in duplicate on the chips. **B.** The chip image. We showed the chip image in triplicate experiments and the enlarged blocks from each chip were on the left.

In the chip assays, biotinylated LfcinB was first probed with *E. coli* proteome chips. DyLight™ 649-labeled streptavidin and DyLight™ 549-labeled anti-His antibodies were then probed with the chips (Figure 3A). The anti-His antibody was used to detect the printed proteins tagged with histidine to represent the relative amount of proteins on the chips, and the majority of the proteins showed strong signals (Figure S1). Because of the strong affinity between biotin and streptavidin, fluorescently labeled streptavidin was used to label LfcinB. The image of proteome chips probed with biotinylated LfcinB in triplicate assays are shown in Figure 3B, and the same enlarged regions from three different chip assays show high reproducibility. ProCAT [17] was used for signal normalization and helped us determine positive hits by the principle of local cut-off, based on the ratio of DyLight™ 649 to DyLight™ 549. By ruling out membrane proteins from the top 5% of the *E. coli* entire proteome, we selected 153 proteins for further analysis (Table 1).

**Table 1:** The top 153 proteins.

Rank 1–26	Rank 27–52	Rank 53–78	Rank 79–104	Rank 105–130	Rank 131–153
HhoB	RplN	ArgH	PurC	YqcA	PtsA
671#8	YhiW	RhaR	AgaY	YrhA	YbbN
YhbN	YegJ	RpsB	YibA	YjdI	XerB
YdaU	YtfI	AmiA	YbeV	YciW	CaiA
BasR	YciT	HyaF	AceK	AtpH	Sun
YifN	YeeL	MdoG	RhaD	YjeJ	SoxR
IlvG	YbaQ	CadA	YffC	HolC	YjhI
RplE	YjdK	YnhE	AdiY	PaaG	YjaE
RplW	DinG	YggF	NrdF	NrdD	YbcQ
YihI	YihS	AroD	HofH	YdeY	WcaL
GldA	CyaA	YgfJ	YhgA	PtsA	RffH
YbaO	YheH	HyaE	PerM	EnvR	YhhI
YjgL	GloA	MetL	YfbR	YbgN	Rob
SoxS	YjbM	IlvL	YcgW	YpdH	YobG
DnaT	SfmH	YibB	YjiV	YhiF	HisA
YdiT	YgiG	RcsB	YibN	YbgE	YbeY
RfaS	YhdG	HolB	YqiA	ThiF	YdhS
CreB	YbcX	GidB	YhaJ	FlgC	YqhC
InfC	DsbG	RecC	HemK	YjeG	YdaW
RfaD	YcgK	RfaZ	Adi	YbeQ	YnjI
YcaO	HemC	Hha	YchA	CbpA	YfbO
YdcC	YrfI	YhaN	GlyQ	YcdN	YfiQ
YbaE	DsbC	FimZ	GlpK	YjiL	YjfW
Smg	GlgS	Ppc	YjeS	YfeF	
SrmB	YlbE	YhjH	YhjC	MalP	
DedD	YehS	YijC	YceC	YefE	

**Figure 4:**
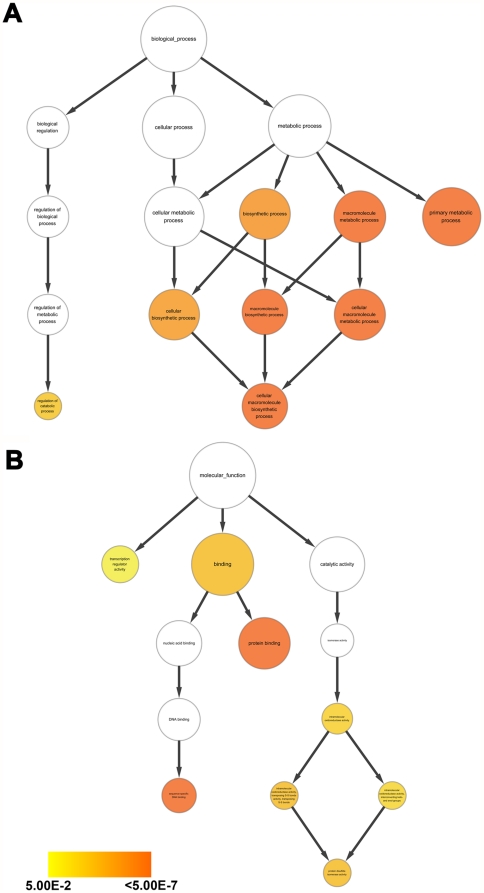
GO analyses of the top 153 identified proteins. GO provides each defined term representing gene product properties. Herein, we showed 2 domains in the figure: **A.** Biological process, **B.** Molecular function. A *p*-value cut-off of 0.05 is used for analyses. The size of the term symbolizes the total gene membership; the size increases with an increasing number of genes. The color depth of each term illustrates the degree of enrichment; the color increases with a decreasing *p*-value. These identified proteins in the chip assays are primarily associated with cellular macromolecular metabolic processes, macromolecular metabolic processes and primary metabolic processes.

To globally classify and analyze the identified proteins in chip assays, we assigned them based on function in GO [18], and the Cytoscape plug-in BiNGO [19] was used to statistically determine enriched proteins of the GO categories in a biological network. From these data, 115 out of 153 candidate proteins in the chip assays were annotated to at least one GO term. As shown in Figure 4, the identified proteins were enriched in the following biological processes: cellular macromolecular metabolic processes, macromolecular metabolic processes, primary metabolic processes, cellular macromolecule biosynthetic processes and macromolecule biosynthetic processes (*p*<10^−4^). These results may explain an earlier finding that LfcinB inhibited bacterial macromolecular biosynthesis [7]. Moreover, more identified proteins were annotated to metabolic processes but fewer to biological regulations. This differentiation suggests that the targets of LfcinB may be highly involved in the general metabolic pathway, but not as regulators. For molecular function (Figure 4B), most of the identified proteins were associated with the term “binding”, in particular “protein binding” (*p*<10^−4^). However, the greatest enrichment was observed in biological processes, not in molecular functions. Specifically, the identified proteins are enriched in downstream metabolic processes.

### Fluorescence polarization assays

**Figure 5:**
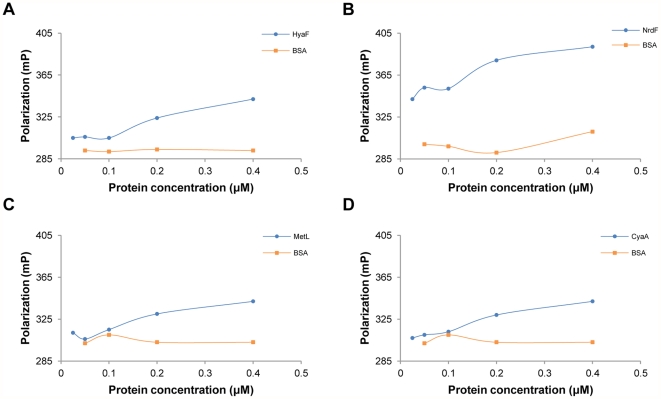
Validation of the interactions between LfcinB and proteins using fluorescence polarization assays. FP assays provide a homogeneous-based environment for a binding test, eliminating immobilization and separation procedures. We used FP assays to conduct screening for protein-peptide interactions. The rotation of fluorescently labeled LfcinB (a small molecule) is faster and hence causes the emitted light to be depolarized. When LfcinB binds to the target proteins, the molecular weight becomes larger and makes the rotation slow. Thus, the degree of polarization becomes higher. In FP assays, the polarization values of the tested proteins were compared with BSA (negative control). The blue circle is a sample and the orange square is BSA. Sixteen out of the 153 proteins have apparent differences from BSA. **A.** HyaF, **B.** NrdF, **C.** MetL and **D.** CyaA are representative FP results.

FP assays, investigations of the binding between two molecules in a homogeneous aqueous environment, were used to validate the identified 153 proteins. These assays have been used to measure various molecular interactions, including protein-protein, protein-peptide and ligand-receptor interactions [20]–[23]. If a fluorophore only binds to a small molecule, such as peptide, the rotation of the complex is fast and the light emits in a different plane of polarization. Therefore, the degree of polarization is low. However, once a fluorophore binds to a large molecule, the bound complex is stimulated with polarized light. The light emits in the same polarized plane, and the complex is maintained in the same plane of polarization, causing an increase in the degree of polarization [22]–[24]. The amount of molecules binding to a fluorophore influences the degree of polarization [24]. In this study, LfcinB was labeled with DyLight™ 549 as a reference value. Once the protein bound to LfcinB, like a fluorophore binds to a large molecule, a high degree of polarization is expected. As shown in Figure 5A–5D, HyaF, NrdF, MetL and CyaA have higher polarization than the negative control (Bovine Serum Albumin, BSA). The degree of polarization remained constant with increasing concentrations of BSA but increased with increasing concentrations of these 4 proteins. These results confirm that LfcinB binds to these 4 representative proteins. Similarly, 12 more proteins (RecC, YnhE, YdaU, YcaO, YqiA, YjbM, YhaN, Ppc, GldA, Adi, MalP and NrdD) were confirmed to bind to LfcinB (data not shown). Therefore, we further analyzed the functions of these 16 proteins. Interestingly, 3 of these proteins (NrdD, NrdF and CyaA) are involved in purine metabolism, and LfcinB had been shown to inhibit DNA and RNA synthesis [7]. One possible explanation of this earlier finding is that LfcinB may attack NrdF, NrdD and CyaA to inhibit DNA synthesis.

### Functional interaction analysis

**Figure 6:**
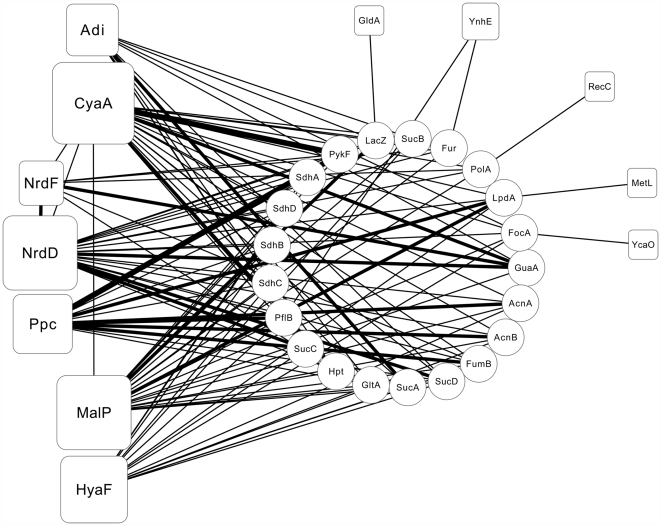
The functional interaction network of the 16 target proteins identified from the fluorescence polarization assays. Each square shape represents the target proteins identified from the FP assays, and round shapes represent the interacting proteins. The EcID was used to find the interacting proteins of these 16 targets. Here, we showed only the proteins that interact with at least 5 targets, and 21 interacting proteins were identified. The thicker line symbolizes that more databases show that they have an interaction between 2 proteins. Seven out of the 16 target proteins, NrdD, CyaA, MalP, Adi, NrdF, Ppc and HyaF, have more interactions and are considered to be hubs.

We further exploited EcID to find the interacting proteins of these 16 identified LfcinB targets to identify the proteins that are indirectly influenced by LfcinB. The EcID integrates information related to functional interactions from several sources, including EcoCyc, KEGG, MINT and IntAct [25]. EcoCyc supplies *E. coli* metabolic pathways, protein complexes and regulatory information; KEGG, MINT and IntAct provide metabolic pathways and protein interaction information. Moreover, the EcID also contains information on protein complexes from high-throughput pull-down assays conducted in *E. coli* and attaches the literature to support potential functional interactions. In this study, we only selected interactions through the experimental mode and not the prediction mode. We also chose the interacting proteins that have functional interactions with at least 5 out of the 16 target proteins identified in the FP assays. We proposed that more target proteins of LfcinB interacting with the same protein may have a larger influence on that protein; conversely, a protein interacting with fewer target proteins of LfcinB is less influenced by LfcinB. Therefore, we chose the interacting proteins that have functional interactions with at least 30 percent of the target proteins (5 out of 16) as a criterion in this study. As shown in Figure 6, 7 targets of LfcinB (MalP, Ppc, CyaA, NrdF, NrdD, Adi and HyaF) were “hubs” that connected many interacting proteins in the network. Thus, the attack of these hubs by LfcinB may have a large influence on *E. coli*. From this analysis, 21 interacting proteins were found to have interactions with at least 5 out of the 16 targets and are thus likely to be indirectly influenced by an LfcinB attack. We further analyzed these 21 interacting proteins. Interestingly, Table S1 shows that 13 out of the 21 proteins are enriched in the TCA cycle. The TCA cycle is a series of enzyme-catalyzed reactions of central importance in living cells that converts carbohydrates, fats and proteins into an available form of energy. These results suggest that LfcinB may indirectly influence energy metabolism in *E. coli*.

**Figure 7:**
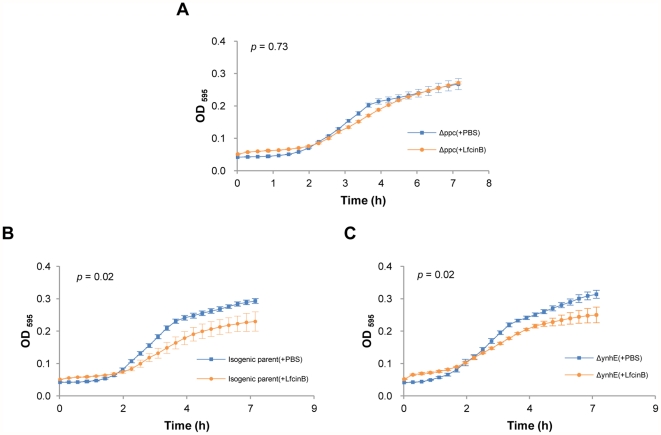
Comparison of the growth inhibition between knockout strains and the isogenic parent strain using knockout assays. We compared the effects of LfcinB on the growth of 16 *E. coli* knockout strains and the isogenic parent strain to examine which gene can escape from the effects of LfcinB. We used PBS (LfcinB dissolving buffer) as the negative control. **A.** Δppc with or without LfcinB have similar growth curves (*p*>0.05). **B.** The isogenic parent strain has a significant difference (*p*<0.05) between treatment with or without LfcinB. **C.** ΔynhE, representing the other 15 knockout strains (*p*<0.05), is similar to the isogenic parent strain. These results indicate that Δppc avoided the LfcinB attack.

### Knockout assays

Knockout strains were designed without a specific gene according to the results of the FP assays. If the lost protein was a target of LfcinB, the knockout strain may avoid the growth inhibition caused by LfcinB and grow with the alternative or redundant pathways. However, knockout assays have a limitation: if the alternative or redundant pathways of the corresponding target protein of the knockout strain are also attacked by LfcinB, the strain will not avoid the growth inhibition caused by LfcinB. Thus, we cannot conclude that the protein is not the target of LfcinB if no growth inhibition is observed in the knockout strain. Knockout assays only provide additional positive confirmations.

The effect of LfcinB on the growth of the 16 *E. coli* knockout strains and their isogenic parent strain were investigated. Interestingly, in the initial part (0–1.5 h) of the growth curves, both the isogenic parent strain and the knockout strains grown with LfcinB had higher growth curves than the strains grown without LfcinB (Figure 7). It is possible that LfcinB served as nutrient for bacteria in the beginning of the assay. Similarly, Ulvatne *et al.* [7] observed that DNA synthesis was increased at the initiation of LfcinB into the medium. In the middle part (2–4 h) of the growth curves, the slopes of both the isogenic parent strain and the knockout strains with LfcinB were lower than the slopes of the strains without LfcinB, indicating that LfcinB started to affect the growth of cells at this time. At the end (after 4 h) of the growth curves, 1 of the 16 knockout strains (Δppc) showed similar OD_595_ value with and without LfcinB (Figure 7A). However, the growth curves of the isogenic parent strain (Figure 7B) as well as the other 15 proteins (ynhE is shown as a representative strain in Figure 7C) with and without LfcinB were obviously different (*p* = 0.02). It appears that LfcinB inhibited the growth of the isogenic parent strain and 15 proteins; however, the growth curves of Δppc were not different with or without LfcinB (*p* = 0.73). These results indicate that the ppc knockout strain successfully protected itself from the LfcinB attack. Moreover, the alternative or redundant pathways of ppc were not influenced by LfcinB; thus, the ppc knockout strain avoided the LfcinB attack. For the other 15 knockout strains, the growth was inhibited by LfcinB because LfcinB may attack the targets and their alternative or redundant pathways simultaneously. Overall, the confidence of Ppc as an LfcinB target was increased through these knockout assays.

### Pyruvate assays

Ppc, phosphoenolpyruvate carboxylase, converts bicarbonate and phosphoenolpyruvate to oxaloacetate. In pyruvate metabolism, phosphoenolpyruvate is converted to either pyruvate or oxaloacetate (Figure S2). When Ppc is inactivated, phosphoenolpyruvate cannot be converted to oxaloacetate, only to pyruvate, which causes an increase in pyruvate concentration. Several studies have reported that the complete knockout of the ppc gene can lead to an increase in the concentration of pyruvate [26], [27], and pyruvate accumulation could inhibit growth [28]. Therefore, one of the mechanisms of LfcinB inhibition of *E. coli* growth may be associated with pyruvate accumulation.

Pyruvate is the simplest alpha-keto organic acid. It is the product of glycolysis and is a key molecule in the network of metabolic pathways that can be converted into a variety of products, such as carbohydrates, fatty acids or energy, amino acids or ethanol. It is involved in several key metabolic processes. To prove that one of the mechanisms of LfcinB action is related to pyruvate metabolism, a pyruvate assay was used to detect the amount of pyruvate in *E. coli* with or without LfcinB. A minimal medium with a sole carbon source, glucose, was used in the pyruvate assays to ensure that pyruvate was generated only via glycolysis. Based on the function of Ppc, we hypothesized that the LfcinB-attacked *E. coli* possibly lost a route of converting phosphoenolpyruvate to oxaloacetate and therefore had an accumulation of pyruvate. In Table 2, the OD_595_ and OD_570_ values represent the amount of *E. coli* and pyruvate, respectively. We observed that the amount of *E. coli* with or without LfcinB as equivalent in the initial stage. After 9 h of incubation, the amount (OD_595_) of *E. coli* with LfcinB was lower than the level of *E. coli* without LfcinB, due to the inhibition of LfcinB; however, the pyruvate (OD_570_) level in *E. coli* with LfcinB was higher than the level in *E. coli* without LfcinB. Thus, these results indicate that *E. coli* with LfcinB has abnormal pyruvate accumulation and this is one reason for the inhibition of *E. coli* growth.

**Table 2:** Pyruvate assay validation.

	*E. coli*	*E. coli *+ LfcinB
Initial value of OD_595_	0.057±0.002	0.058±0.001
Value of OD_595_ after 9 h incubation	0.325±0.011	0.267±0.011
Value of pyruvate (OD_570_) after 9 h incubation	1.976±0.084	2.526±0.338

*Note*. We used pyruvate assay kit to detect the pyruvate levels of *E. coli* with or without LfcinB treatment. The *E. coli* with and without LfcinB had equivalent amounts in the initial stage. After an incubational time of 9 h, we found that *E. coli* with LfcinB had more pyruvate than the *E. coli* without LfcinB, even though it had lower amount of *E. coli*.

## Discussion

### A rapid, reliable and high-throughput tool to study antimicrobial peptides

The proteome chip is a useful technology that has been applied to various types of research [10], [11], [15], [16]. In this study, we used this tool to study the interactions between the defense systems of multi-cellular organisms and bacteria. Understanding the mechanisms of how these natural antimicrobial peptides targeted and disrupted the complex regulatory network within the cells may provide clues for the development of new strategies and compounds to against bacteria effectively. By using proteome chips, we can easily obtain proteins that were possible targets of LfcinB. We then conducted bioinformatic analyses (ProCAT and GO analyses) to process the chip results. ProCAT was used to normalize the chip signals and obtain a reliable protein list and GO analyses were used to classify the protein list.

### Limitations of knockout assays

To validate the chip-identified proteins that bind to LfcinB, FP assays were used to study the interactions between proteins and LfcinB. FP assays have been used to identify the inhibitors of the p53-DM2 protein-protein interaction [29]. Through FP assays, we obtained a list of 16 proteins that bind to LfcinB. We used an additional validation method (knockout assays) to identify these select proteins and observe the physiological condition of the bacteria. With this assay, we identified only 1 target protein of LfcinB, Ppc. However, many proteins exhibiting specific dose-dependent polarization values in the FP assays, such as NrdF (ribonucleoside-diphosphate reductase 2, β subunit dimer), were not confirmed to be associated with LfcinB activity in the knockout assays. One possible explanation for this disparity is gene redundancy, a limitation of knockout assays. When several genes perform the same function and have similar structures, they may be attacked simultaneously by LfcinB. In this case, a single gene knockout could not avoid the LfcinB attack; therefore, growth was still inhibited by LfcinB. For example, NrdF has a similar function as NrdB [30], [31]; they both convert ribonucleotide to deoxynucleotide, providing the precursors for DNA synthesis and repair [32], [33]. When NrdF is inactive, NrdB action can replace the function of NrdF. NrdF and NrdB also have similar structures [30]. Our chip results showed that both NrdF and NrdB were targets of LfcinB. Although NrdB was not in the top 153 proteins, it still had a strong binding affinity with LfcinB in chip assays. The growth curve of the nrdF knockout mutant with LfcinB was significant inhibited compared to the growth curve of the nrdF knockout mutant without LfcinB, indicating that the nrdF knockout strain cannot avoid the LfcinB attack because nrdB was also attacked by LfcinB. Although NrdF was not validated in knockout assays, it may still be a target of LfcinB. These data show the limitations of knockout assays.

### The EcID analysis

The EcID was downloaded and processed to uncover proteins that were highly related to the targets. According to the network, 7 out of the 16 FP identified proteins were hubs. These hubs have different functions, including pyruvate metabolism, purine/pyrimidine metabolism, amino acid metabolism and carbohydrate metabolism. Three out of the 7 hubs, NrdF, NrdD and CyaA, were associated with purine metabolism. NrdF and NrdD belong to the ribonucleotide reductase family and supply the materials for DNA synthesis and repair. CyaA is an adenylate cyclase that catalyzes the conversion of ATP to cyclic-AMP. Although they are all involved in purine metabolism, NrdF and NrdD have a more direct relationship with DNA synthesis than CyaA. As mentioned previously, the synthesis of DNA and RNA is inhibited by LfcinB [7], which may be due to an LfcinB attack of NrdF and NrdD.

We analyzed not only the hubs but also interacting proteins. The majority of the proteins that interact with at least 5 out of the 16 identified proteins in the FP assays (SdhD, AcnA, AcnB, SdhA, SdhB, LpdA, SucA, SucD, SucC, FumB, SdhC, SucB and GltA) were involved in the TCA cycle (13 out of 21); a minority of these proteins participated in purine/pyrimidine metabolism (PolA, PykF, GuaA and Hpt) or pyruvate metabolism (PykF, PflB and LpdA). Of the proteins that interact with at least 6 out of the 16 identified proteins in the FP assays, 6 interacting proteins were retained in the network. Four out of these 6 interacting proteins (LpdA, SucD, FumB and SucC) were related to the TCA cycle. Of the proteins that interact with at least 7 out of the 16 identified proteins in the FP assays, PflB was the only interacting protein in the network. PflB is an enzyme that catalyzes the conversion of pyruvate into formate and acetyl-CoA (Figure S2). When PflB was inactive, it stopped the normal pyruvate conversion, which may lead to an increase in pyruvate levels [34]. Therefore, these data may also explain the pyruvate accumulation seen when *E. coli* is under the LfcinB attack.

### LfcinB and pyruvate accumulation

Ppc is one of the direct targets of LfcinB, as seen in the results from the chip assays, FP assays and knockout assays. However, PflB is an indirect target of LfcinB, according to the functional interaction analysis (EcID). PflB was the only protein that interacts with at least 7 out of the 16 identified proteins in the FP assays, suggesting that once the directly targeted proteins were attacked by LfcinB, PflB may be inactive because it was associated with those proteins. Phosphoenolpyruvate, the upstream metabolite of pyruvate, has only two downstream pathways. The first pathway forms oxaloacetate through the catalysis of Ppc, and the other pathway forms pyruvate. PflB further converts pyruvate to formate and acetyl-CoA (Figure S2). Therefore, when Ppc was inactive, it stopped phosphoenolpyruvate from being converted to oxaloacetate and only allowed the conversion to pyruvate; when PflB was inactive, it stopped pyruvate from being converted to formate. Although their mechanisms are different, the inactivation of Ppc and PflB both cause pyruvate accumulation.

### Conclusion

This study used high-throughput *E. coli* proteome chips to find potential intracellular mechanisms of action for antimicrobial peptides. Chip and FP assay results indicate that LfcinB has multiple targets. Knockout and pyruvate assays showed that one of the mechanisms of LfcinB action is related to pyruvate accumulation. These findings suggest that the proteome chip is a useful and novel tool for studying the intracellular targets and mechanisms of antimicrobial peptides. We expect a wide application of this approach for studying the interactions of other antimicrobial peptides and antibacterial agents.

## Materials and Methods

### Fabrication of an *E. coli* proteome chip

To fabricate the proteome chips, *E. coli* K-12 proteins were expressed and purified in a high-throughput fashion. The procedure was modified from Chen *et al.* [9]. Briefly, *E. coli* protein expression clones fused with a 6×-His tag, provided by Dr. Mori [35], were first incubated with glass beads (Biospec Products Inc.) in 2× LB medium containing 30 µg/ml chloramphenicol in 96 DeepWell™ plates (Nunc) at 37°C overnight. The overnight cultures were then diluted with 2× LB that contains 30 µg/ml chloramphenicol to the OD_595_ value of 0.1. When the cells grew to an OD_595_ value of 0.7–0.9, 500 µM of isopropyl β-D-thiogalactoside (IPTG) was added to induce protein expression at 37°C for ∼3.5 h. The cultures were then harvested by centrifugation at 3220 g for 5 min at 4°C, and the pellets were stored at −80°C before purification.

To purify the proteins, the frozen cell pellets were thawed on ice and resuspended at 4°C in 40 µl of lysis buffer, consisting of 50 mM NaH_2_PO_4_, 300 mM NaCl, 30 mM imidazole, CelLyticB (Sigma-Aldrich), 1 mg/ml lysozyme, 50 units/ml benzonase, proteinase inhibitor cocktail (Sigma-Aldrich), 1 mM phenylmethanesulfonyl fluoride (PMSF) and Ni-NTA Superflow resins (Qiagen). After a 2.5 h incubation on a plate shaker at 4°C, the mixtures were transferred into 96-well filter plates (Nunc) and washed with wash buffer I (50 mM NaH_2_PO_4_, 300 mM NaCl, 20% glycerol, 20 mM imidazole and 0.1% Tween 20, pH 8) 5 times and wash buffer II (50 mM NaH_2_PO_4_, 150 mM NaCl, 30% glycerol, 30 mM imidazole and 0.1% Tween 20, pH 8) 5 times. Finally, the proteins were eluted with elution buffer (50 mM NaH_2_PO_4_, 150 mM NaCl, 30% glycerol, 300 mM imidazole and 0.1% Tween 20, pH 7.5). When the purified proteins were prepared, they were printed in duplicate along with BSA, serum proteins, antibodies and histone as negative controls or landmarks on Fullmoon slides by ChipWriter Pro (Bio-Rad) with 48 pins in a 4°C cold room. After printing, the chips were immobilization at 4°C for at least 8 h and then stored at −80°C before utilization.

### The influence of biotinylated LfcinB, LfcinB and biotin on *E. coli* growth

The *E. coli* K-12 MG1655 strain was first inoculated into LB medium and incubated at 37°C with shaking for 16 h. After incubation, the culture was diluted to approximately 10^7^ CFU/ml using LB medium. Then, 22.4 µM of N-terminal biotinylated LfcinB (Kelowna Inc.), LfcinB (Kelowna Inc.) or biotin (Thermo) were individually added to the diluted cultures in a 96-well plate. PBS was used as a negative control. Finally, each compound or PBS with *E. coli* was incubated at 37°C with shaking and the growth curves were observed by measuring the OD_595_ value every 20 min for 7 h 20 min in a microplate reader (Synergy 2, BioTek®).

### *E. coli* proteome chip assays with LfcinB

The chip was first blocked with 1% BSA. Ten nM of N-terminal biotinylated LfcinB with 1% BSA was probed with the chip in a hybridization chamber on a 3D shaker at room temperature for 1 h. The chip was then washed 3 times with Tris-buffered saline-Tween 20 (TBS-T). DyLight™ 549-labeled anti-His antibody (abcam®) and DyLight™ 649-labeled streptavidin (Thermo) were then probed on the chip at room temperature for 30 min. Finally, the chip was washed 4 times with TBS-T in an orbital shaker. The chip was dried by centrifugation at 201 g and then scanned with a microarray scanner (Axon GenePix® 4000B) after being rinsed with distilled water.

### Bioinformatics analyses of the chip assay results

For image processing of chip assay results, Genepix Pro 6.0 was used to align the protein spot of each individual protein and then all aligned protein image signals were exported as a text file. The text file was analyzed by ProCAT [17], a signal normalization method. The relative ability of LfcinB to bind to each protein was estimated by the ratio of the fluorescence intensity of LfcinB to the fluorescence intensity of an anti-His antibody after the normalization. All the bioinformatics analyses were performed by Perl 5.0.

For the gene enrichment analysis of the identified proteins, the BiNGO software, a plug-in for Cytoscape, was used [19]. Because the gene annotation file of *E. coli* was not included in the default settings, the annotation file was downloaded from the GO Website (http://www.geneontology.org/) and imported while the GO was in progress. A *p*-value cut-off of 0.05 was used for the analyses.

### Fluorescence polarization assays

Before the assays, LfcinB was labeled with DyLight™ 549 and tested proteins were quantified with a Pierce® bicinchoninic acid (BCA™) protein assay (Thermo). To label the LfcinB, DyLight™ 549-NHS ester and LfcinB (68∶1, mole/mole) were mixed in PBS at room temperature for 1 h, a D-Salt™ polyacrylamide desalting column (Thermo) was used to remove excess dye and the labeling efficiency of the eluted solution was calculated.

After blocking the 96-well black plate with 1% BSA at room temperature for 1 h, a two-fold serial dilution of proteins from 0.4 µM to 0.025 µM was prepared in the assay buffer (PBS, 0.005% BSA and 0.01% Triton X-100) and added to the plate. Serially diluted BSA (0.4 µM, 0.2 µM, 0.1 µM and 0.05 µM) was also used as a negative control. After calculating the labeling efficiency, 100 nM of fluorescence-labeled LfcinB, used as a tracer, was incubated with protein or BSA at room temperature for 1 h. The degree of polarization of each well was detected by a microplate reader, using an excitation wavelength of 540 nm and an emission wavelength of 590 nm with a dichroic mirror of 570 nm.

### Functional interaction analysis

The EcID was used for functional interaction network analyses of the identified proteins from the FP assays. Briefly, the entity and pair files were downloaded from EcID. For data pre-processing, the pair file was filtered by removing the protein-protein interaction pairs that were based on prediction mode, such as phylogenetic profiles or gene neighborhoods. After pre-processing, the identified proteins were mapped to their EcID IDs and the pair file was scanned to recognize which proteins will interact with the identified proteins. Cytoscape [36] was used to generate the functional protein interaction network, and it visualized the identified proteins with their interacting partners. All of the intermediate procedures were completed by Perl 5.0.

### Knockout assays

The *E. coli* K-12 BW25113 (isogenic parent) strain and the 16 knockout strains were obtained from the *E. coli* Genetic Resources at the Coli Genetic Stock Center (CGSC) at Yale University. The strains were inoculated into LB medium, and incubated at 37°C with shaking. After 16 h of incubation, the cultures were diluted to approximately 10^7^ CFU/ml with LB medium. Thirty-two µM of LfcinB were then added to the cultures in a 96-well plate. PBS was used as a negative control. Cultures with LfcinB or PBS were then incubated at 37°C with shaking in a microplate reader and the growth was observed by measuring the OD_595_ value every 20 min for 7 h 20 min.

### Pyruvate assays

To confirm one of the mechanisms of LfcinB action in pyruvate metabolism, a minimal medium was chosen; because general LB medium contains many carbon sources, including sugars, alcohols and organic acids, this minimal medium allowed glucose to be the sole carbon source in the pyruvate assays. Minimal medium consisted of 6 g/l NaH_2_PO_4_, 3 g/l K_2_HPO_4_, 0.5 g/l NaCl, 0.12 g/l MgSO_4_, 1 g/l NH_4_Cl, 0.01 g/l CaCl_2_ and 5 g/l glucose. A 0.22 µm filter (Millex®GS, Millipore) was used to filter the minimal medium. *E. coli* MG1655 was first inoculated into the minimal medium and incubated at 37°C for 16 h with shaking as a preculture. The preculture was diluted 100-fold with the minimal medium in 96-well plate, and 32 µM of LfcinB were added. The mixture was incubated with shaking in a microplate reader at 37°C, and the absorbance was measured after 9 h. Finally, the mixture was harvested by centrifugation at 3452 g for 5 min at 4°C, and each supernatant was removed. The pellets were then stored at −80°C.

To extract the pyruvate in *E. coli*, hot water extraction was used, a procedure adapted from Canelas *et al.* [37]. Boiling water was poured into the pellet and it was immediately vortexed. The mixture was then placed in a water bath at 95–100°C for 15 min and cooled on ice. When the extraction was complete, a pyruvate assay kit (BioVision, K609-100) was used to detect the pyruvate level in the extracts. A reaction mixture, containing a pyruvate assay buffer, a pyruvate probe and an enzyme mix, was added to each pyruvate extract. The mixtures were then incubated for 30 min at room temperature to allow pyruvate oxidization by pyruvate oxidase and the solution to change color to red. Finally, the OD_570_ of the mixtures was measured by a microplate reader to detect the color intensity.

## Supporting Information

Figure S1 **The quality of an** ***E. coli*** **proteome chip.** The chip image showed the quality of a protein chip by probing DyLight™ 549-labeled anti-His antibody. The majority of the proteins show strong signals.
(TIF)

Figure S2 **Partial pyruvate pathway.** Phosphoenolpyruvate and bicarbonate are converted to oxaloacetate by Ppc. Phosphoenolpyruvate is converted to pyruvate by PykFA. Pyruvate is converted to formate and acetyl-CoA by PflB.
(TIF)

Table S1 **The pathways of the interacting proteins identified from EcID.**
(DOC)
