# Supplementary figures and images for: Identification of Lactoferricin B Intracellular Targets Using an Escherichia coli Proteome Chip
Source: PLoS One. 2011 Dec 2;6(12):e28197. doi: 10.1371/journal.pone.0028197 (PMC3229523; doi:10.1371/journal.pone.0028197)

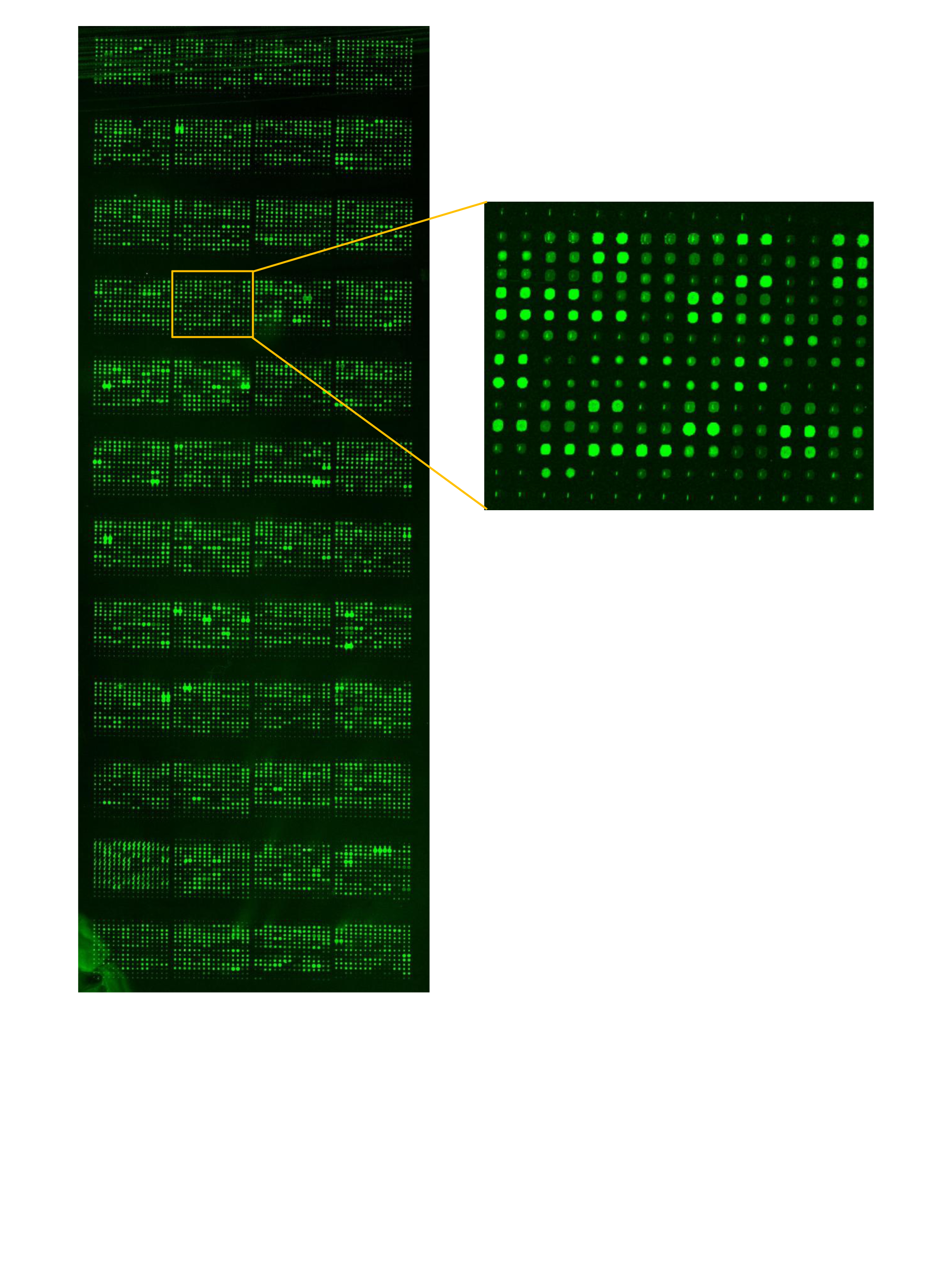

Supplement: Figure S1 — The quality of an E. coli proteome chip. The chip image showed the quality of a protein chip by probing DyLight™ 549-labeled anti-His antibody. The majority of the proteins show strong signals. (TIF) [file pone.0028197.s001.tif]

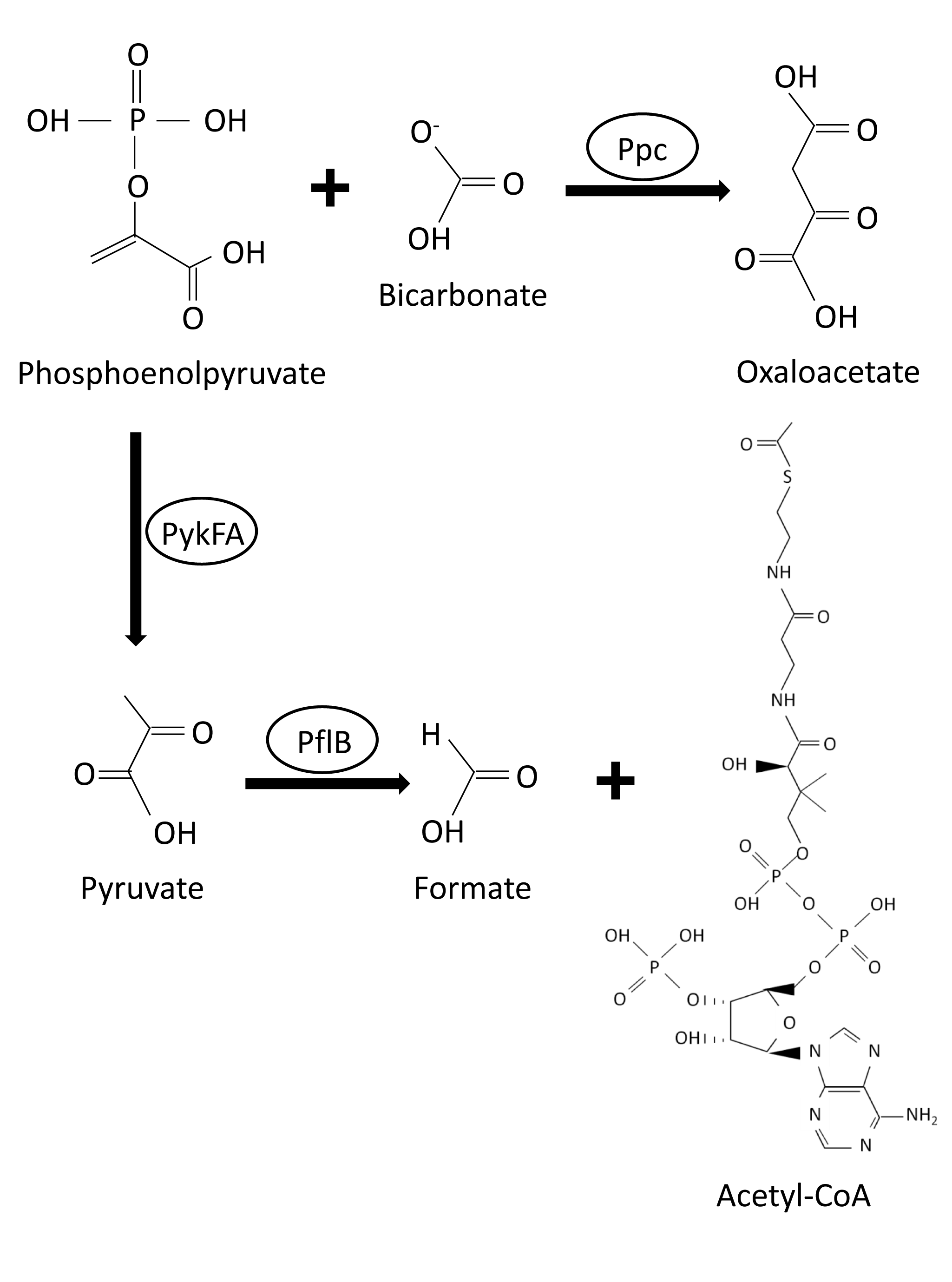

Supplement: Figure S2 — Partial pyruvate pathway. Phosphoenolpyruvate and bicarbonate are converted to oxaloacetate by Ppc. Phosphoenolpyruvate is converted to pyruvate by PykFA. Pyruvate is converted to formate and acetyl-CoA by PflB. (TIF) [file pone.0028197.s002.tif]
