# Supplementary material for: Identification of Lactoferricin B Intracellular Targets Using an Escherichia coli Proteome Chip
Source: PLoS One. 2011 Dec 2;6(12):e28197. doi: 10.1371/journal.pone.0028197 (PMC3229523; doi:10.1371/journal.pone.0028197)
Supplement: Table S1 — The pathways of the interacting proteins identified from EcID. (DOC) [file pone.0028197.s003.doc]

**Table S1. The pathways of the interacting proteins identified from EcID.**

| **Gene** | **Name** | **Pathways** |
| --- | --- | --- |
| polA | DNA polymerase I | Purine metabolism |
|  | 5' --> 3' polymerase | Pyrimidine metabolism |
|  | 5' --> 3' and 3' --> 5' exonuclease | DNA replication |
|  |  | Base excision repair |
|  |  | Nucleotide excision repair |
| pykF | Pyruvate kinase | Glycolysis/ Gluconeogenesis |
|  |  | Purine metabolism |
|  |  | Pyruvate metabolism |
| pflB | Pyruvate formate-lyase | Pyruvate metabolism |
|  |  | Propanoate metabolism |
|  |  | Butanoate metabolism |
| sdhD | Succinate dehydrogenase membrane protein | TCA cycle |
|  |  | Oxidative phosphorylation |
|  |  | Toluene degradation |
|  |  | Butanoate metabolism |
|  |  | Carbon fixation pathways |
| acnA | Aconitate hydratase | TCA cycle |
|  |  | Glyoxylate and dicarboxylate metabolism |
|  |  | Carbon fixation pathways |
| acnB | Bifunctional aconitate hydratase 2 | TCA cycle |
|  | 2-methylisocitrate dehydratase | Glyoxylate and dicarboxylate metabolism |
|  |  | Carbon fixation pathways |
| sdhA | Succinate dehydrogenase flavoprotein | TCA cycle |
|  |  | Oxidative phosphorylation |
|  |  | Toluene degradation |
|  |  | Butanoate metabolism |
|  |  | Carbon fixation pathways |
| sdhB | Succinate dehydrogenase iron-sulfur protein | TCA cycle |
|  |  | Oxidative phosphorylation |
|  |  | Toluene degradation |
|  |  | Butanoate metabolism |
|  |  | Carbon fixation pathways |
| lacZ | β-galactosidase monomer | Galactose metabolism |
|  |  | Other glycan degradation |
|  |  | Sphingolipid metabolism |
| lpdA | E3 monomer | Glycolysis/ Gluconeogenesis |
|  |  | TCA cycle |
|  |  | Glycine, serine and threonine metabolism |
|  |  | Valine, leucine and isoleucine degradation |
|  |  | Pyruvate metabolism |
| guaA | GMP synthetase | Purine metabolism |
| sucA | 2-oxoglutarate dehydrogenase E1 component | TCA cycle |
|  |  | Lysine degradation |
|  |  | Tryptophan metabolism |
| sucD | Succinyl-CoA synthetase, α subunit | TCA cycle |
|  |  | Propanoate metabolism |
|  |  | C5-branched dibasic acid metabolism |
|  |  | Carbon fixation pathways |
| sucC | Succinyl-CoA synthetase, β subunit | TCA cycle |
|  |  | Propanoate metabolism |
|  |  | C5-branched dibasic acid metabolism |
|  |  | Carbon fixation pathways |
| fumB | Fumarase B monomer | TCA cycle |
|  |  | Carbon fixation pathways |
| sdhC | Succinate dehydrogenase membrane protein | TCA cycle |
|  |  | Oxidative phosphorylation |
|  |  | Toluene degradation |
|  |  | Butanoate metabolism |
|  |  | Carbon fixation pathways |
| sucB | Dihydrolipoyltranssuccinylase | TCA cycle |
|  |  | Lysine degradation |
| hpt | Hypoxanthine phosphoribosyltransferase | Purine metabolism |
| gltA | Citrate synthase | TCA cycle |
|  |  | Glyoxylate and dicarboxylate metabolism |
| fur | Fur family transcriptional regulator |  |
| focA | Formate transporter |  |
